# Detection of oncogenic mutations in paired circulating tumor DNA and circulating tumor cells in patients with hepatocellular carcinoma

**DOI:** 10.1016/j.tranon.2021.101073

**Published:** 2021-04-26

**Authors:** Zhouhong Ge, Jean C.A. Helmijr, Maurice P.H.M. Jansen, Patrick P.C. Boor, Lisanne Noordam, Maikel Peppelenbosch, Jaap Kwekkeboom, Jaco Kraan, Dave Sprengers

**Affiliations:** a Departments of Gastroenterology and Hepatology, Erasmus MC-University Medical Center, Wytemaweg 80, Rotterdam 3015 CN, The Netherlands; b Departments of Medical Oncology, Erasmus MC-University Medical Center, Rotterdam, The Netherlands

**Keywords:** Circulating tumor DNA, Circulating tumor cells, TERT promoter mutations, Macrovascular invasion, Hepatocellular carcinoma, ctDNA, circulating tumor DNA, CTC, circulating tumor cells, ddPCR, droplet digital PCR, HCC, hepatocellular carcinoma, NASH, non-alcoholic steatohepatitis, NAFLD, non-alcoholic fatty liver disease, *TERT*, telomerase reverse transcriptase, VAF, variant allele frequency, MVI, macrovascular invasion

## Abstract

**Background and aims:**

Circulating tumor cells (CTCs) or circulating tumor DNA (ctDNA) may be used for diagnostic or prognostic purposes in patients with hepatocellular carcinoma (HCC). We aim to determine whether CTCs or ctDNA are suitable to determine oncogenic mutations in HCC patients.

**Methods:**

Twenty-six mostly advanced HCC patients were enrolled. 30 mL peripheral blood from each patient was obtained. CellSearch system was used for CTC detection. A sequencing panel covering 14 cancer-relevant genes was used to identify oncogenic mutations. *TERT* promoter C228T and C250T mutations were determined by droplet digital PCR.

**Results:**

CTCs were detected in 27% (7/26) of subjects but at low numbers (median: 2 cells, range: 1–15 cells) and ctDNA in 77% (20/26) of patients. Mutations in ctDNA were identified in several genes: *TERT* promoter C228T (77%, 20/26), *TP53* (23%, 6/26), *CTNNB1* (12%, 3/26), *PIK3CA* (12%, 3/26) and *NRAS* (4%, 1/26). The *TERT* C228T mutation was present in all patients with one or more ctDNA mutations, or detectable CTCs. The *TERT* C228T and *TP53* mutations detected in ctDNA were present at higher levels in matched primary HCC tumor tissue. The maximal variant allele frequency (VAF) of ctDNA was linearly correlated with largest tumor size and AFP level (Log10). CtDNA (or *TERT* C228T) positivity was associated with macrovascular invasion, and positivity of ctDNA (or *TERT* C228T) or CTCs (≥ 2) correlated with poor patient survival.

**Conclusions:**

Oncogenic mutations could be detected in ctDNA from advanced HCC patients. CtDNA analysis may serve as a promising liquid biopsy to identify druggable mutations.

## Highlights

• In paired analysis CTCs were detected in 27% and ctDNA in 77% of HCC patients.
• The *TERT* promoter mutation C228T was present in all patients with one or more ctDNA mutations, or detectable CTCs.
• CtDNA (or *TERT* C228T) positivity was associated with macrovascular invasion and poor survival of advanced HCC patients.

## Introduction

Hepatocellular carcinoma is the 4th leading cause of cancer related death worldwide [1]. Treatments options for advanced disease are limited. Most patients are diagnosed at a late stage and their median survival is less than 2 years [2]. In advanced patients tumor tissue is usually obtained by needle biopsies, which are invasive and does not capture the heterogeneity of the whole tumor. Liquid biopsies, including circulating tumor cells (CTCs) and cell-free DNA (ctDNA), have been suggested to allow identification of tumor-derived mutations for use in diagnosis and rational application of targeted therapies while patient burden associated with such sampling is minimal.

CTCs are cells that have detached from a primary or secondary tumor and enter circulation [3], and are regarded as seeds for metastasis. There has been a technical challenge to capture them due to the extremely low frequency, estimated ≤ 1 CTC/ml of blood in billions of blood cells. CellSearch is a standardized and validated method to detect EpCAM^+^ CTCs from epithelial tumors not including HCC [4]. Nevertheless, many studies show that CellSearch identifies CTCs in 16–67% HCC patients in early or late stage [5], [6], [7], [8], [9], [10], [11], [12]. Other enrichment methods include size-based enrichment [13], density-based RosetteSep negative selection [14], magnetic negative selection [15], flowcytometric detection [16] and microfluidic enrichment [17]. Most CellSearch studies report prognostic value of CTC numbers in HCC [5,[8], [9], [10],12]. The CTC-positive rate in peripheral blood was associated with tumor size, serum alpha-fetoprotein level (AFP), vascular invasion and overall survival [5,^10^,11]. A preoperative CTC positivity is a predictor for tumor recurrence in HCC patients after surgery [8,9]. The increase in postoperative CTC counts was significantly associated with the macroscopic tumor thrombus and shorter overall survival [12]. Moreover, to help guide treatment decisions, for instance to initiate or switch targeted therapy, it is important to determine acquisition of somatic mutations. Because of the risks associated with repeated tumor biopsies, it is attractive to use liquid biopsies for this purpose. Mutational analysis in CTCs can be performed by DNA sequencing [11,18]. However, CTCs found by numerous methods have often not been analyzed for DNA mutations in HCC, likely due to limited numbers and inefficiency of single cell isolation techniques.

Circulating tumor DNA (ctDNA) is the fraction of cell-free DNA that is derived from primary or metastatic tumors. The fraction of circulating mutant DNA fragments is very small, sometimes less than 0.01% [19], compared to circulating wildtype DNA fragments, making it difficult to detect and quantify [20]. The development of next generation sequencing (NGS), especially deep sequencing, and droplet digital PCR (ddPCR) have facilitated the identification of genetic variants in ctDNA. Chan et al. was the first to report analysis of ctDNA by shotgun sequencing of plasma samples from HCC patients in early stage. The ctDNA concentration, determined by single nucleotide variants (SNVs) analysis, was found to range from 2.1% to 53% before surgery and from 0.4% to1.3% after surgery [21]. HCC-associated mutations (e.g. *TP53, TERT, CTNNB1, APC, EGFR, MET, ARID1A*) were detected in ctDNA by NGS sequencing in advanced [22,23] and operable HCC [24,25]. The presence of somatic mutations in ctDNA before surgery could predict microvascular invasion in resectable HCC patients [24,25]. Moreover, postoperative residual ctDNA was an independent risk factor for recurrence and poor disease-free survival in HCC patients [26]. Very recently, TERT promotor mutated ctDNA was described to be a prognostic biomarker for HCC [27,28], but this observation requires further validation.

Therefore, in clinical practice it becomes more and more relevant that we have access to easy and reliable diagnostic tools for detection of oncogenic mutations to help guide treatment decisions. Hence, in this study, we used paired samples of CTCs and ctDNA to explore feasibility to detect oncogenic mutations in HCC patients.

## Material and methods

### Patients and blood collection

A total of 26 HCC-patients were enrolled in the study between May 2018 and July 2019*.* Peripheral blood samples (30 mL) from each patient were collected before any anti-tumor treatment and were collected into Cellsave tubes (Menarini Silicon Biosystems, Huntington Valley, PA). Matched FFPE primary tumor tissue biopsies were obtained from department of Pathology in Erasmus MC. The study was approved by the local ethics committee (METC), Erasmus MC, Rotterdam (METC number: 17-238), and written informed consent was obtained from each patient. The clinical characteristics of the 26 patients are summarized in Table 1.

**Table 1:** Patient characteristics.

	HCC patients (*n* = 26)
Gender	
Male	23
Female	3
Age at sampling (years)	
< 60	6
≥ 60	20
Mean ± SD	67 ± 11.6
Race	
Caucasian	23
Asian	2
African	1
Etiology	
No known liver disease	9
Hepatitis B/C	3/2
Alcohol-related liver disease	7
NASH/NAFLD*	4/1
Cirrhosis	
No	11
Yes	15
BCLC stage (A/B/C)	3/5/7
Size of largest lesion (cm)	
< 10	7
≥ 10	19
Mean ± SD	13 ± 5.2
Tumor number	
1	17
> 1	9
Macrovascular invasion	
No	12
Yes	14
AFP level (µg/L)	
< 20	12
20–400	3
> 400	11
Treatment	
Surgery	1
TACE/SIRT⁎⁎	5
Systemic therapy (Sorafenib)	10
BSC⁎⁎⁎	10

⁎ NASH, non-alcoholic steatohepatitis; NAFLD, non-alcoholic fatty liver disease.

⁎⁎ TACE, transarterial chemoembolization; SIRT, selective internal radiation therapy.

⁎⁎⁎ BSC, best supportive care.

### Plasma separation and ctDNA extraction

Blood samples were processed within 24 h after collection. Blood was first centrifuged at 1700 g for 10 min to separate plasma and blood cells. The separated plasma was centrifuged at 12,000 g for another 10 min to remove cellular debris. The plasma was collected and aliquoted in vials per 2 ml and stored at −80 °C until further processing. ctDNA was isolated from 440 µL to 4 mL (median 3.95 mL) plasma using QIA-amp Circulating Nucleic Acid kit (Qiagen, Venlo, the Netherlands) and eluted in 30 µL elution buffer. CtDNA concentrations were determined by Qubit™ 1X dsDNA HS Assay kit (Thermo Fisher scientific, Waltham, MA) using 2 µL of ctDNA.

### Enrichment of CTCs and single cell isolation

7.5 mL whole blood was used to detect EpCAM^+^ CTCs by CellSearch system (Menarini Silicon Biosystems). The cells were isolated by EpCAM positive selection using the CellSearch Circulating Tumor Cell kit ® and defined as CD45^−^CK(8/18/19)^+^ DAPI^+^_._ The stained and scanned CTCs were scored blindly by two certified researchers. Single CTC were isolated from the CellSearch cartridge with the VyCAP cell puncher system (VyCAP, Enschede, The Netherlands) using an isolation chip, which consists of 6400 microwells [29]. Single cells were subjected to whole-genome amplification (WGA) by the Ampli1 WGA kit (Menarini Silicon Biosystems) and the DNA quality of the WGA-products was determined with the WGA Quality control kit (VyCAP) according to manufactures instructions.

### Amplification of ctDNA

The extracted ctDNA was pre-amplified for the targets *TERT* C228T and *TERT* C250T by PCR. In brief, a pre-amp reaction mix (total volume: 8 µL) was prepared for each target using: 4 µL Tagman PreAMP Master Mix (Thermo Fisher Scientific), 2 µL ctDNA sample and 2 µL of 100× diluted *TERT* C228T-113 or *TERT* C250T-113 assay (Bio-Rad, Herucles, CA). Then PCR was performed using the following cycle conditions: 1 cycle of 10 min at 95 °C,15 cycles of 15 s at 95 °C and 4 min at 60 °C and finally hold at 4 °C. After pre-amplification PCR 72 µL ultrapure DNAse/RNAse free H_2_O (Thermo Fischer Scientific/Gibco) was added to the reaction for a final volume of 80 µL.

### Droplet digital PCR (ddPCR)

For the quantification of the *TERT* C228T and C250T mutations in ctDNA from HCC patients, ddPCR was performed using the Naica Crystal PCR system (Stilla Technologies, Beverly, MA). Prior to the ddPCR, pre-amplified ctDNA samples were diluted 20× for every 0.5 ng of ctDNA input in the pre-amplification reaction to prevent saturation with DNA copies of the ddPCR Sapphire chips (Stilla Technologies) with copies. Based on the resulting amount of target copies of the first ddPCR (low sample concentration input), a second ddPCR (high sample concentration input) was performed with an input of at least 2500 target copies. For each target the following ddPCR reaction mix was prepared: 1 µL of diluted pre-amplified ctDNA sample, 14 µL ddPCR™ supermix for probes (Bio-Rad), 2.8 µL of 5 M Betaine (Sigma Aldrich, Darmstadt, Germany), 1 µL of 28 mM EDTA (Thermo Fisher Scientific), 1.4 µL of *TERT* C228T-113 or *TERT* C250T-113 assay (Bio-Rad) [30], 2.8 µL of 1 µM Fluorescein (VWR, Radnor, PA) and finally ultrapure DNAse/RNAse free H_2_O (Thermo Fischer Scientific/Gibco) was added to bring up the total volume to 28 µL. Then 26 µL of the reaction mix was then loaded unto the Sapphire chips (Stilla Technologies) and ddPCR was performed using the following cycle conditions: 1 cycle of 10 min at 95 °C, 50 cycles of 30 s at 96 °C and 1 min at 62 °C, 1 cycle of 10 min at 98 °C and finally hold at 4 °C. The Sapphire Chips were scanned using Naica Prism3 system with default exposure times for the FAM-labeled mutant probe (50 ms) and the HEX-labeled wildtype probe (250 ms). Then, data was analyzed using the Stilla Crystal Miner v2.4.0.3 software and thresholds were set based on positive and negative controls for each mutation assay.

To exclude false positive samples, all ctDNA were analyzed at low and high concentrations. An increase in sample concentration should result in an elevated number of mutant copies compared to the lower concentrated sample. If mutant copies were detected and no increase was observed in the higher concentrated sample of the same patient, the patient was regarded negative for either the *TERT* C228T or C250T mutation. Finally, a minimum threshold of 5 detected mutant copies was established to discriminate between *TERT* mutation positive and negative patients. The variant allele fraction (VAF) was calculated as the proportion of ctDNA harboring the variant in a background of wild-type cell-free DNA.

### Targeted next-generation sequencing (NGS)

Sequencing was performed by Ion semiconductor sequencing on the Ion Torrent S5XL Next generation sequencing (NGS) system using the Oncomine ctDNA Assay with molecular barcoding loaded on Ion 540 chips. Experiments were performed according to the manufacturer's protocol (Thermo Fisher Scientific/Life Technologies). Since there is no customized oncopanel for HCC in EMC, we chose the Oncomine™ Colon ctDNA panel (Thermo Fisher/Life Technologies) which contains the most frequently mutated driver genes in HCC. This panel comprises of 14 colon cancer-related genes (*TP53, CTNNB1, APC*, BRAF, AKT1, PIK3CA, *EGFR*, ERBB2, KRAS, NRAS, GNAS, MAD4, MAP2K1, FBXW7) covering > 240 mutational hotspots. Sample input ranged between 2.8 ng and 20.7 ng DNA in 13 µl for the sequence reaction. Basecalling was performed using the Ion Torrent Suite Software 5.10 plugin (Thermo Fisher Scientific/Life Technologies) according the manufactures protocol with default basecalling settings. Variant calling was performed using the Ion Torrent Suite software and Variant caller plugin (Thermo Fisher Scientific/Life Technologies) and the variant caller parameters can be found in Supplementary Table 5. Additionally, the following post-variant caller filters were used to eliminate false positive variants: only known hotspot variants were selected when detected in at least 3 independent mutant molecules, with a variant allele frequency of at least 0.2% and the total sequencing depth at that the variant position (wildtype and mutant) was at least 500 independent (wildtype and mutant) molecules. Data were being made publically available online at the European Genome-Phenome Archive (ega-archive.org).

### Statistical analysis

Spearman's rank correlation test for nonparametric data was used to analyze the correlation between two factors. Kaplan-Meier analysis is used to evaluate the survival differences. Clinical parameters in different groups were compared using Chi-Square test. Cox regression model was used for univariate and multivariate analysis. Statistical analysis was performed using Graphpad Prism 8.0 or IBM SPSS statistics 25. *P* value less than 0.05 was considered statistically significant (*P < 0.05; **P < 0.01; ***P < 0.001; ****P < 0.0001).

A detailed description of other methods is provided in the *Supplemental information*.

## Results

### Description of study cohort

In total 26 patients diagnosed with HCC were enrolled. The patient characteristics are summarized in Table 1. With the exception of one patient who could undergo curative surgery, all patients were treated with palliative therapy because of comorbidity, severity of liver disease and/or advanced tumor stage. Male individual accounted for the majority (88%, 23/26) of subjects included. The median diagnostic age of enrolled HCC patients was 68.5 years (ranged from 40 to 85). Most of patients are Caucasian (88%, 23/26). In 9/26 (35%) patients there was no known liver disease, almost half of patients had alcohol-related liver disease or NASH/NAFLD (46%, 12/26), and a small percentage of patients had a chronic hepatitis B/C infection (19%, 5/26). Cirrhosis existed in 15 patients (58%). The largest tumor diameter was more than or equal to 10 cm in 73% (19/26) of patients. Nine patients (35%) had multiple malignant tumor foci. Macrovascular invasion was detected in 14 patients (54%). Fifteen of 26 pts (58%) were cirrhotic (BCLC stage A/B/C: 3/5/7, respectively). Elevated AFP (> 400 µg/L) was seen in 11 patients (42%).

### CTC count and mutation profiling of CTC

**Fig. 1:**
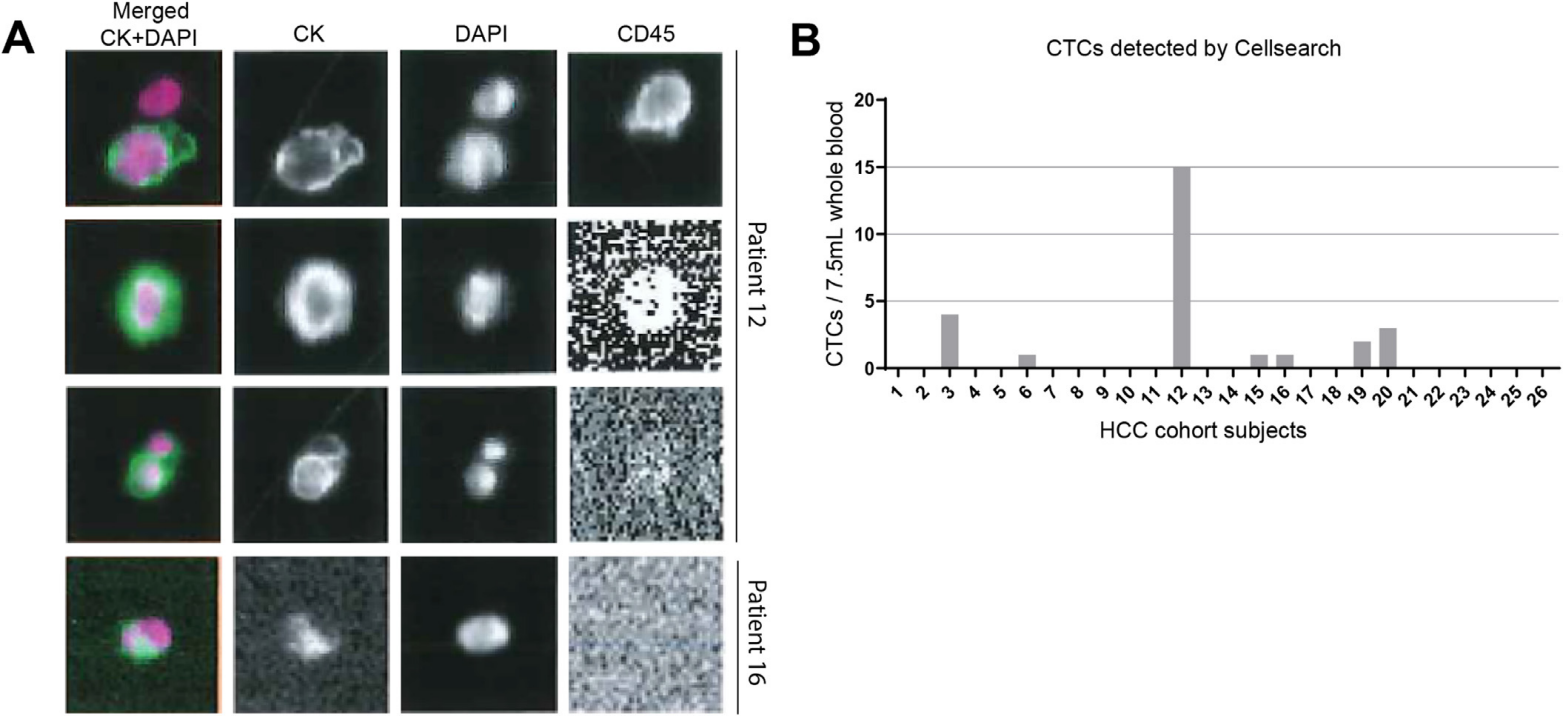
**CTC counts in advanced HCC patients**. (A) Representative images of CellSearch show EpCAM enriched CTCs which are CK^+^DAPI^+^CD45^−^. (B) The number of CTCs in individual HCC patient identified by the CellSearch system (*n* = 26).

As CellSearch based on EpCAM positive selection is the only U.S. FDA-approved technique for CTC detection, an important prerequisite for a diagnostic tool to be implemented in clinical practice, we used the CellSearch system to detect CTCs in HCC patients. For CTC analysis, twenty-six patients were enrolled and 7.5 mL peripheral blood from each patient was used. The number of CTCs detected by CellSearch ranged from 1 to 15/7.5 mL blood and 27% (7/26) of patients were CTC-positive (Fig. 1A and B**).** Among CTC-positive patients, 4 out of 7 had ≥ 2 CTCs. Using the Vycap system, we managed to isolate 8 single CTC (53% recovery) from the CellSearch enriched CTC fraction from the patient that had 15 CTCs (patient 12). We did whole genome amplification for each single CTC and only one CTC had good quality of the WGA and could be analyzed for the oncogenic mutations by ddPCR and NGS sequencing. However, we did not find any mutations in this CTC. Importantly, we also did not detect any oncogenic mutation in ctDNA from the plasma of this patient.

Since CellSearch is dependent on EpCAM expression by CTC, we analyzed the expression of EpCAM on tumor cells using tissue microarrays with cores of resected tumors from 109 early-stage HCC patients (surgery candidates as described previously in [31,32]) by immunohistochemistry (IHC) staining. EpCAM expression in tumor tissue was found in only 7% (7/109) of these HCC patients (**Figs. S1A–S1D**).

Collectively, we detected CTCs in a minority of our cohort of mostly advanced HCC patients and we could not isolate sufficient single CTC cell for mutational analysis.

### Mutational landscape in HCC by ctDNA profiling

**Fig. 2:**
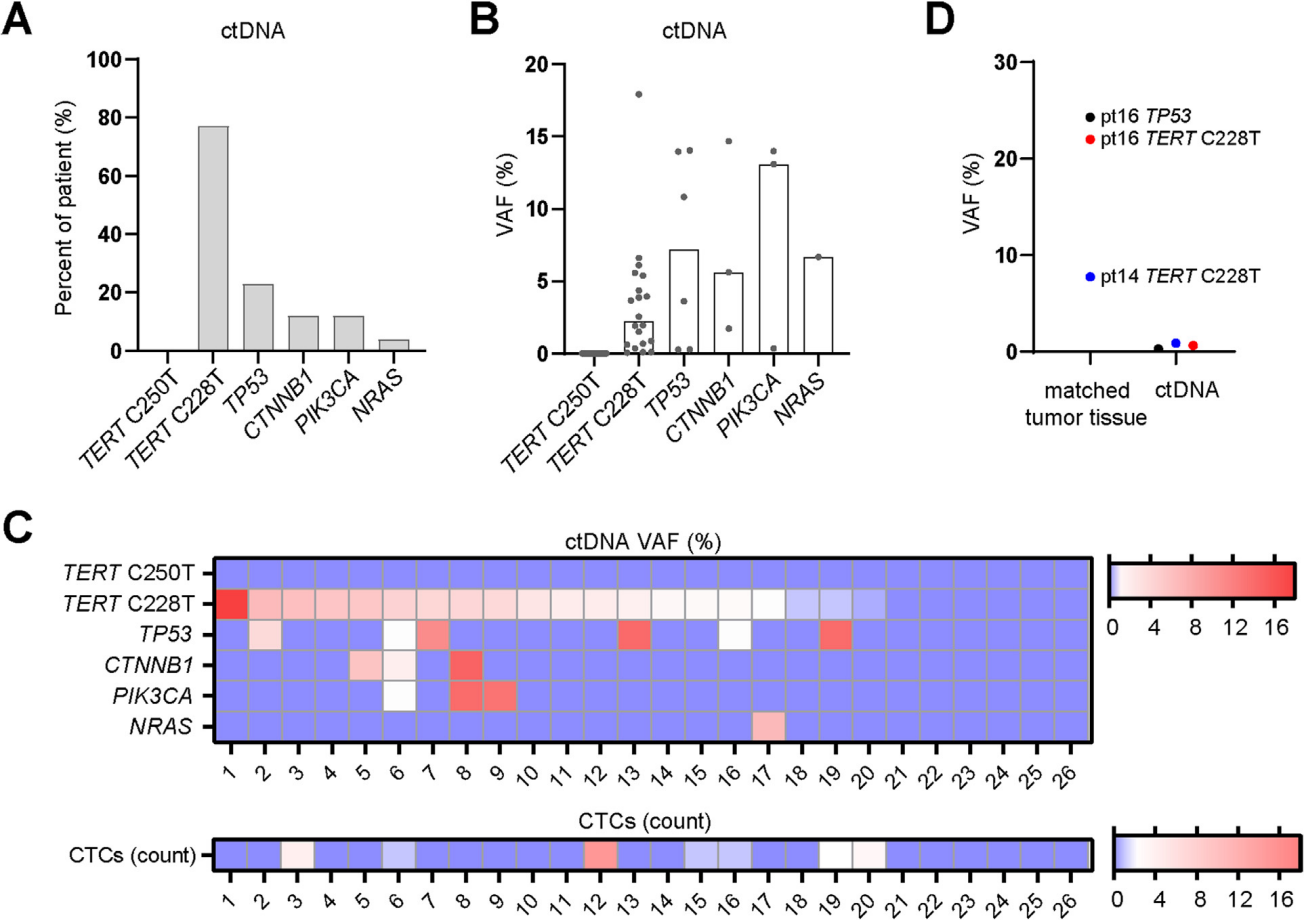
**Mutational landscape in advanced HCC by ctDNA profiling**. (A) The percentages of HCC patients that were positive for each mutated gene detected in ctDNA. TERT mutations were detected by ddPCR and other mutations were detected by NGS sequencing. (B) The VAF for each mutated gene in each HCC patient. Bars show median value of VAF. (C) Heat map shows ctDNA VAF and CTC count in each of the 26 HCC patients. VAF, variant allele frequency. (D) The VAF of mutated genes in ctDNA and matched tumor tissue.

As *TERT* promoter mutations are the most common mutations present in around 60% of HCC patients [33], we analyzed *TERT* promoter mutations C228T and C250T by ddPCR in order to estimate the ctDNA amount in blood from HCC patients. We additionally analyzed somatic mutations with a NGS panel covering 14 oncogenic genes. The amount of ctDNA that we isolated ranged from 7 to 282.4 ng per ml of plasma (mean 45.52 ng/ml plasma) (**Fig. S2A**). The majority of patients (77%, 20/26) had at least one mutation in ctDNA, only (23%, 6/26) had no detectable mutations (**Tables S1 and S2**). The *TERT* C228T mutation (20/26, 77%) was the most frequently detected mutation whereas the *TERT* C250T mutation was absent in all patients (Fig. 2A). *TP53* (6/26, 23%) was the second most frequently detected mutation, followed by *CTNNB1* (3/26, 12%), *PIK3CA* (3/26, 12%) and *NRAS* (4%, 1/26) (Fig. 2A). The median VAF for ctDNA mutations are 2.76% (range: 0.3−17.92%) for *TERT* C228T, 7.21% (range: 0.28−14.05%) for *TP53*, 5.63% (range: 5.63−14.68%) for *CTNNB1* and 13.09% (0.35−13.99%) for *PIK3CA* (Fig. 2B). Interestingly, the *TERT* C228T mutation was present in all patients with one or more ctDNA mutations, and this mutation was detectable in all patients of which CTCs were detectable (Fig. 2C). However, ctDNA maximal VAF did not correlate with CTC count (**Fig. S2B**).

Subsequently, we explored the concordance of these mutations in ctDNA and matched tumor tissue. There were two matched primary tumor biopsies available (patients 14 and 16) for mutational analysis, and in those tissues *TERT* C228T and *TP53* (hotspot p.R249S) mutations were present at much higher frequency in tumor compared to ctDNA (Fig. 2D).

Collectively, ctDNA mutations can be detected in our HCC patients and the mutational landscape in ctDNA matches the published HCC mutation landscape acquired by bulk sequencing of tumor tissues [34].

### ctDNA positively correlated with macrovascular invasion, tumor size and AFP level

**Fig. 3:**
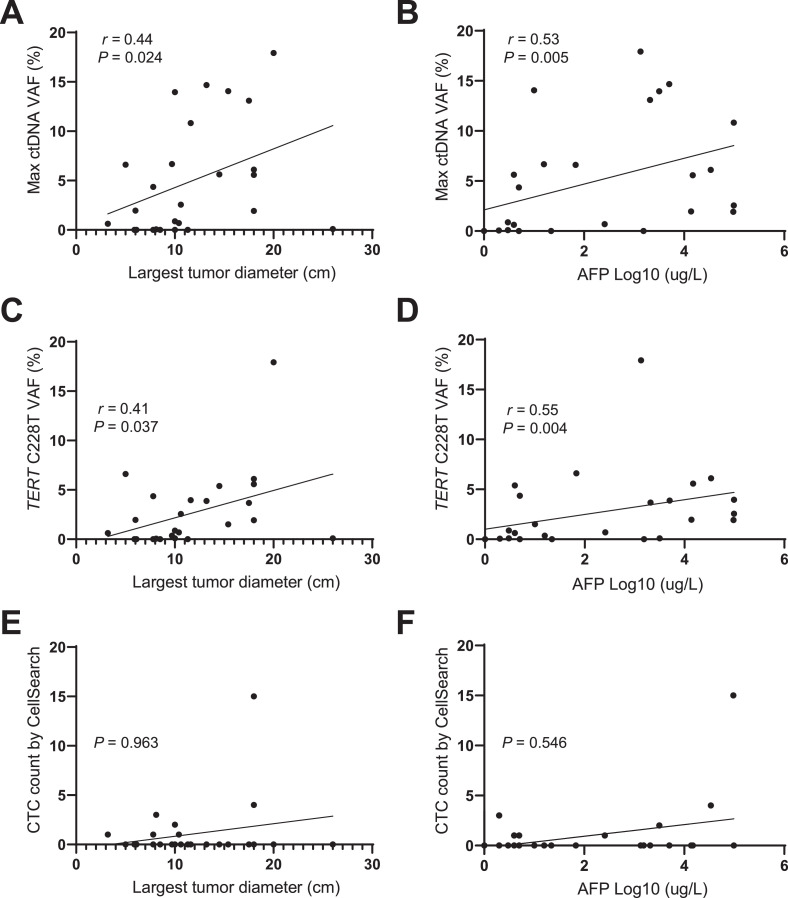
**ctDNA positively correlated with macrovascular invasion, tumor size and AFP level**. (A–D) Linear correlations between maximal ctDNA VAF or *TERT* C228T VAF and largest tumor diameter or AFP level. (E,F) Absence of correlations between CTC count and tumor size or AFP (Log10). Spearman's rank correlation test is used to analyze the correlation between two factors. VAF, variant allele frequency.

We analyzed whether ctDNA status or CTC count correlated with clinicopathologic parameters known to be associated with prognosis. Clinical parameters in different groups were compared using Chi-Square test. We found that ctDNA positivity (or *TERT* C228T positivity) correlated with macrovascular invasion (MVI) as determined on imaging with CT/MRI, whereas for CTCs we did not observe such correlation (Table 2). We next performed a linear regression analysis to further explore the correlation between clinicopathologic parameters and ctDNA VAF or CTC counts. Results showed that maximal ctDNA VAF was positively correlated with the size of the largest tumor (*r* = 0.44, *P* = 0.024) and AFP level (*r* = 0.53, *P* = 0.005) (Fig. 3A and B). Also *TERT* C228T VAF significantly correlated with largest tumor diameter (*r* = 0.41, *P* = 0.037) and AFP (Log10) level (*r* = 0.55, *P* = 0.004) (Fig. 3C and D). In contrast, the CTC count did not show such correlations with largest tumor diameter or AFP (Log10) level (Fig. 3E and F).

**Table 2:** Comparison of the clinical parameters between classified ctDNA or CTC groups.

Clinicopathologic parameters	ctDNA pos (*n* = 20)	ctDNA neg (*n* = 6)	*P* value	CTC ≥ 2 < *n* = 4)	CTC <2 < *n* = 22)	*P* value
	N (%)	N (%)		N 1%)	N 1%)	
Age, years						
< 60	6 (23.1)	1(3.8)	1.000	2 (7.7)	5 (19.2)	0.287
≥ 60	14 (53.8)	5 (19.2)		2 (7.7)	17 (65.4)	
Cirrhosis						
No	9 (34.6)	2 (7.7)	1.000	1 (3.8)	10 (38.5)	0.614
Yes	11 (42.3)	4 (15.4)		3 (11.5)	12 (46.2)	
Tumor size						
< 10	4 (15.4)	3 (11.5)	0.293	0 (0)	7 (26.9)	0.546
≥ 10	16 (61.5)	3 (11.5)		4 (15.4)	15 (57.7)	
Tumor number						
1	14 (53.8)	3 (11.5)	0.628	3 (11.5)	14 (53.8)	1.000
> 1	6 (23.1)	3 (11.5)		1 (3.8)	8 (30.8)	
Macrovascular						
invasion						
No	6 (23.1)	6 (23.1)	0.004	0 (0)	12 (46.2)	0.100
Yes	14 (53.8)	0 (0)		4 (15.4)	10 (38.5)	
AFP (ng/mL)						
< 20	8 (30.8)	4 (15.4)	0.365	1 (3.8)	11 (42.3)	0.598
≥ 20	12 (46.2)	2 (7.7)		3 (11.5)	11 (42.3)

### ctDNA positivity or CTC count correlated with HCC patient survival

**Fig. 4:**
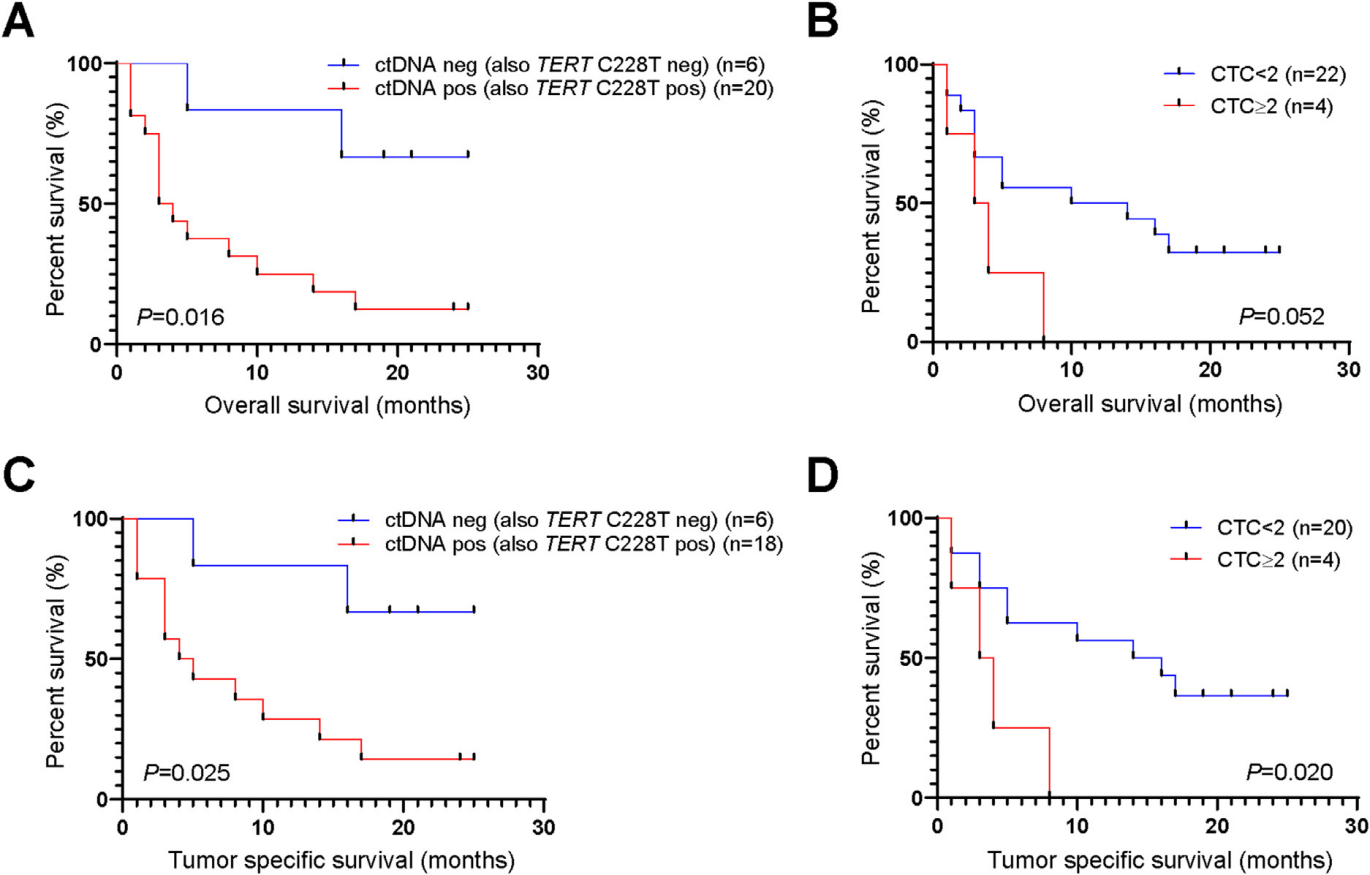
**ctDNA positivity or CTC count correlated with HCC patient survival**. (A) Patients with positive ctDNA (or *TERT* C228T) and negative ctDNA (or *TERT* C228T) demonstrated differentiated overall survival. (B) Survival curve for patients with CTC count < 2 and ≥ 2. Kaplan–Meier analysis is used to evaluate the survival differences. *P* < 0.05 is considered significant.

We next investigated whether ctDNA status or CTC count correlated with overall survival (OS) or tumor specific survival. Patients with one or more ctDNA mutations (*TERT* C228T positive patients) had a significantly worse overall survival than patients without ctDNA mutations (median OS 3 vs 17.5 months, *P* = 0.016) (Fig. 4A). Patients with CTC ≥ 2 tended to have a worse overall survival than patients with CTC ≤ 2 (median OS 3.5 vs 5 months, *P* = 0.052) (Fig. 4B). Moreover, patients that were ctDNA+ (or *TERT* C228T+) or with CTC ≥ 2 had significantly worse tumor-specific survival (Fig. 4C and D). We performed cox regression analysis to reveal the risk factors for overall survival. Univariate analysis revealed that ctDNA positivity and MVI were risk factors for overall survival, whereas multivariate analysis demonstrated that these two factors were dependent on each other (Table S3). This result was validated in chi-square test, which shows that ctDNA positivity was significantly associated with MVI (*P* = 0.004) (Table 2).

## Discussion

Liquid biopsies, including CTCs and ctDNA, may potentially be used to identify tumor-derived mutations for HCC diagnosis and to guide decisions with regard to targeted therapies. In this study, we compare CTCs and ctDNA from the same patient for oncogenic mutational profiling in a cohort of mostly advanced HCC.

EpCAM^+^ CTCs have previously been detected in 28−35% of advanced HCC patients in Western cohorts by CellSearch [5, 6,11], which is lower than the frequencies reported in Asian cohorts (EpCAM^+^ CTCs: 44−74%) focusing mostly on resectable HCC patients [8,^10^,12]. In line with these findings, we found CTCs in 27% of a Western cohort of advanced HCC patients using this method. Moreover, in 43% (3/7) of CTCs+ patients only 1 CTC/7.5 ml blood could be detected. Such low detection rate limits the use of this technique for mutational analysis. There can be several explanations for the low CTC detection rate: CTCs are simply not present (the frequency is < 1 in 7.5 mL blood), or they cannot be detected by this assay because the expression of capture (EpCAM) and/or identification (CK) markers is too low or lacking in a subset of patients. EpCAM was expressed in tumors of only 7% of HCC patients in our IHC analysis, and moreover has been reported to be downregulated during epithelial-mesenchymal transition (EMT) [35,36]. The frequency of EpCAM expression in our western HCC cohort was lower compared to Asian cohorts in which its frequency ranges from 15% to 56% of patients [37], [38], [39], [40], [41], [42], [43]. This might be due to the difference in etiology of liver disease. EpCAM expression is reported to correlate with HBV infection [37,^38^,42]. However, in our cohort only 8% HBV+ patients were EpCAM^+^ (2/24). Although IHC may be less sensitive to detect EpCAM expression compared to immunomagnetic capture with ferrofluids and the CellSearch enhanced aggregation technique, our data emphasize the limitations of EpCAM-based detection of circulating tumor cells in HCC for mutational analysis due to the low frequency of CTCs in our patient cohort. Several other techniques for CTC detection have been described [4], but they have as far as we know not been FDA approved, which hampers widespread implementation in clinical practice.

In contrast, HCC-associated mutations could be detected in 77% of patients using ctDNA. In our cohort, 77% of patients have *TERT* promoter mutations, 23% have *TP53* and 12% have *CTNNB1* or *PIK3CA* mutations. Moreover, in all ctDNA+ patients the *TERT* promotor C228T mutation was present, whereas one or more additional oncogenic mutation(s) was/were present in 50% of these patients. Overall the observed frequencies of mutations are consistent with NGS results of HCC tumors [34]: Totoki et al. detected in HCC histology: 55% *TERT* promoter mutations, 31% *TP53*, 31% *CTNNB1* and 1% *PIK3CA* mutations, respectively (a comparison of all oncogenic mutations analyzed is provided in supplementary Table 4). Our data are also in line with other studies reporting that oncogenic alteration in ctDNA can be detected in 57% (8/14) – 88% (181/206) of advanced HCC patients [22,23]. Discrepancies in reported frequencies may be related to differences in clinical characteristics of the populations studied since genomic distinction has been associated with HCC risk factors [22,23]; we have included mostly Caucasian patients with unknown or alcoholic liver disease, whereas the above mentioned studies include more patients with viral etiology of their liver disease. In our experimental design there can be two possible explanations for ctDNA negativity: (1) the absence of any targeted mutation in the primary tumor, (2) the targeted oncogenic mutation was present in the primary tumor but it could not be detected in blood. To illustrate, recently, TERT promotor mutation in paired plasma and tissue biopsy were concordant in 21/34 patients (62%). In the 12 of 13 non-concordant samples, the TERT mutation was found in tumor but not in plasma [27]. In agreement with other studies [26,^44^–46], we found mutation concordance between ctDNA and primary tumor tissue in two patients but since in our clinic routine tumor biopsies are uncommon in advanced HCC patients (in context of cirrhosis) our dataset of matched tissues is too small to draw conclusions.

In our cohort presence of ctDNA was associated with clinicopathologic parameters of advanced disease. Maximal ctDNA VAF correlated with tumor size and AFP level, and ctDNA positivity was associated with macrovascular invasion. Moreover we showed that ctDNA positivity was associated with overall and tumor-specific survival in our HCC patients and *TERT* C228T mutation alone is a significant predictor of survival. These findings are in line with recent findings by others, that have correlated TERT promotor mutation in plasma with macrovascular invasion and overall survival [27,28]. The ddPCR technique used in our study for TERT promoter mutation analysis is a relatively easy, fast and affordable assay, that can be used in clinical care for detection of this biomarker, for instance as an additional tool in the early detection of HCC, as was suggested by others [47,48]. Previously, ctDNA mutations in early-stage HCC patients have been associated with microvascular invasion and recurrence by others [44,45]. ctDNA positivity in postoperative plasma predicted poor disease-free survival after tumor resection in early HCC-patients [26].

Somatic mutations detected in ctDNA can guide treatment decisions with regard to targeted therapies. Mutation analysis of *NRAS/KRAS/BRAF, PIK3CA* and *CSF-1R* in plasma has been applied in a phase 2 clinical trial to assess response to a mitogen-activated protein kinase (MEK) inhibitor (refametinib) in advanced HCC patients [49]. Ikeda et al. [22] evaluated 14 patients with advanced HCC and detected druggable mutations in 79% of patients. Based on their findings patients were treated with customized therapies. A patient with a *CDKN2A*-inactivating and a *CTNNB1*-activating mutation received palbociclib (CDK4/6 inhibitor) and celecoxib (COX-2/Wnt inhibitor) treatment and found declined des-gamma-carboxy prothrombin (DCP) level after 2 months of treatment. Another patient with a *PTEN*-inactivating and a *MET*-activating mutation received sirolimus (mechanistic target of rapamycin inhibitor) and cabozantinib (*MET* inhibitor) and demonstrated signs of clinical efficacy. The ease-of-use of mutational analyses using ctDNA as opposed to the more cumbersome CTC isolation allows for repeated, longitudinal analysis for prognostic and therapeutic decision making in HCC patients. In our cohort, we detected druggable mutations in 5 patients, including 3 patients with *CTNNB1*, 3 patients with *PIK3CA* and 1 patient with *NRAS* mutations.

Our study has several limitations: (1) we have a relatively small cohort of patients; (2) as mentioned, we lack sufficient matched primary tumor tissue biopsies for comparison; and (3) we did not perform paired leukocyte DNA sequencing thus we could not fully exclude that *TP53* mutations were derived from leukocyte DNA, because somatic variants in *TP53* found in ctDNA could be derived from clonal hematopoiesis [50,51]; (4) the ctDNA panel that we used is limited and does not contain all known actionable somatic alterations (e.g. mutation in *TSC1*/*TSC2*, amplification in *EGFR* and *MET*).

In conclusion, we compared CTC and ctDNA for detecting tumor mutations in a cohort of mostly advanced HCC patients. CTCs as detected by Cell Search are present in low frequency and it is challenging to isolate single CTC for mutational analysis. In contrast, ctDNA is detectable in 77% of our HCC patients. CtDNA detection is associated with known prognostic markers for disease survival. Our study illustrates that analysis of ctDNA may serve as a liquid biopsy to identify druggable mutations in advanced HCC patients.

## Author contributions statement

**Zhouhong:** Conceptualization; Data curation; Formal analysis; Funding acquisition; Investigation; Methodology; Resources; Software; Validation; Visualization; Roles/Writing – original draft; Writing – review & editing.

**Jean C.A. Helmijr**: Data curation; Formal analysis; Methodology; Software; Validation; Visualization; Writing – review & editing.

**Maurice P.H.M. Jansen:** Conceptualization; Formal analysis; Methodology; Software; Visualization; Writing – review & editing.

**Patrick P.C. Boor:** Investigation; Methodology; Supervision.

**Lisanne Noordam:** Visualization; Writing – review & editing.

**Maikel Peppelenbosch:** Conceptualization; Investigation; Methodology; Project administration; Supervision.

**Jaap Kwekkeboom:** Conceptualization; Investigation; Methodology; Resources; Supervision; Writing – review & editing.

**Jaco Kraan:** Conceptualization; Investigation; Methodology; Project administration; Resources; Software; Supervision; Validation; Visualization; Writing – review & editing.

**Dave Sprengers:** Conceptualization; Funding acquisition; Investigation; Methodology; Project administration; Resources; Software; Supervision; Validation; Visualization; Writing – review & editing.

## Declaration of Competing Interest

The authors declare that they have no known competing financial interests or personal relationships that could have appeared to influence the work reported in this paper.

## Appendix Supplementary materials

Image, application 1
