# Supplementary material for: Detection of oncogenic mutations in paired circulating tumor DNA and circulating tumor cells in patients with hepatocellular carcinoma
Source: Transl Oncol. 2021 Apr 26;14(7):101073. doi: 10.1016/j.tranon.2021.101073 (PMC8100622; doi:10.1016/j.tranon.2021.101073)
Supplement: Supplementary file 1 [file mmc1.pdf]

Supplemental Material and Methods

Immunohistochemistry (IHC)

To estimate the frequency of HCC patients with EpCAM expressing tumors cells, expression of EpCAM was determined on tumor cells using tissue microarrays with cores of normal liver tissue (obtained from livers of multi-organ donors), tumors and paired tumor-free liver tissues from 109 HCC-patients by immunohistochemistry (cohort and tissue micro-arrays were described previously) [1, 2]. The TMAs were immunohistochemically stained by the department of pathology of Erasmus MC. IHC was performed with an automated, validated and accredited staining system (Ventana Benchmark ULTRA, Ventana Medical Systems, Tucsen, AZ, USA) using Optiview universal DAB detection Kit (#760-700). In brief, following deparaffinization and heat-induced antigen retrieval the tissue samples were incubated according to their optimized time with CD155. Incubation was followed by hematoxylin II counter stain for 12 minutes and then a blue coloring reagent for 8 minutes according to the manufactures instructions (Ventana). The immunohistochemically stained TMAs were then scanned using NanoZoomer 2.0HT (Hamamatsu) and scored blindly by two researchers, based on the intensity of staining (0[none],1[low], 2[intermediate], 3[strong]) and the frequency of positive tumor cells or hepatocytes (A[<10%], B[10-50%], C[50-90%], D[>90%]). The score per core was calculated by multiplying the intensity by the frequency of positive cells (A=0.1, B=0.3, C=0.7 and D=1), and then the average score per tissue was calculated by taking the average of the three scores.

References

1. Sideras K, Bots SJ, Biermann K, et al. Tumour antigen expression in hepatocellular carcinoma in a low-endemic western area. Br J Cancer 2015;112:1911-20.
2. Sideras K, Biermann K, Verheij J, et al. PD-L1, Galectin-9 and CD8(+) tumor-infiltrating lymphocytes are associated with survival in hepatocellular carcinoma. Oncoimmunology 2017;6:e1273309.

Supplementary Figure 1.

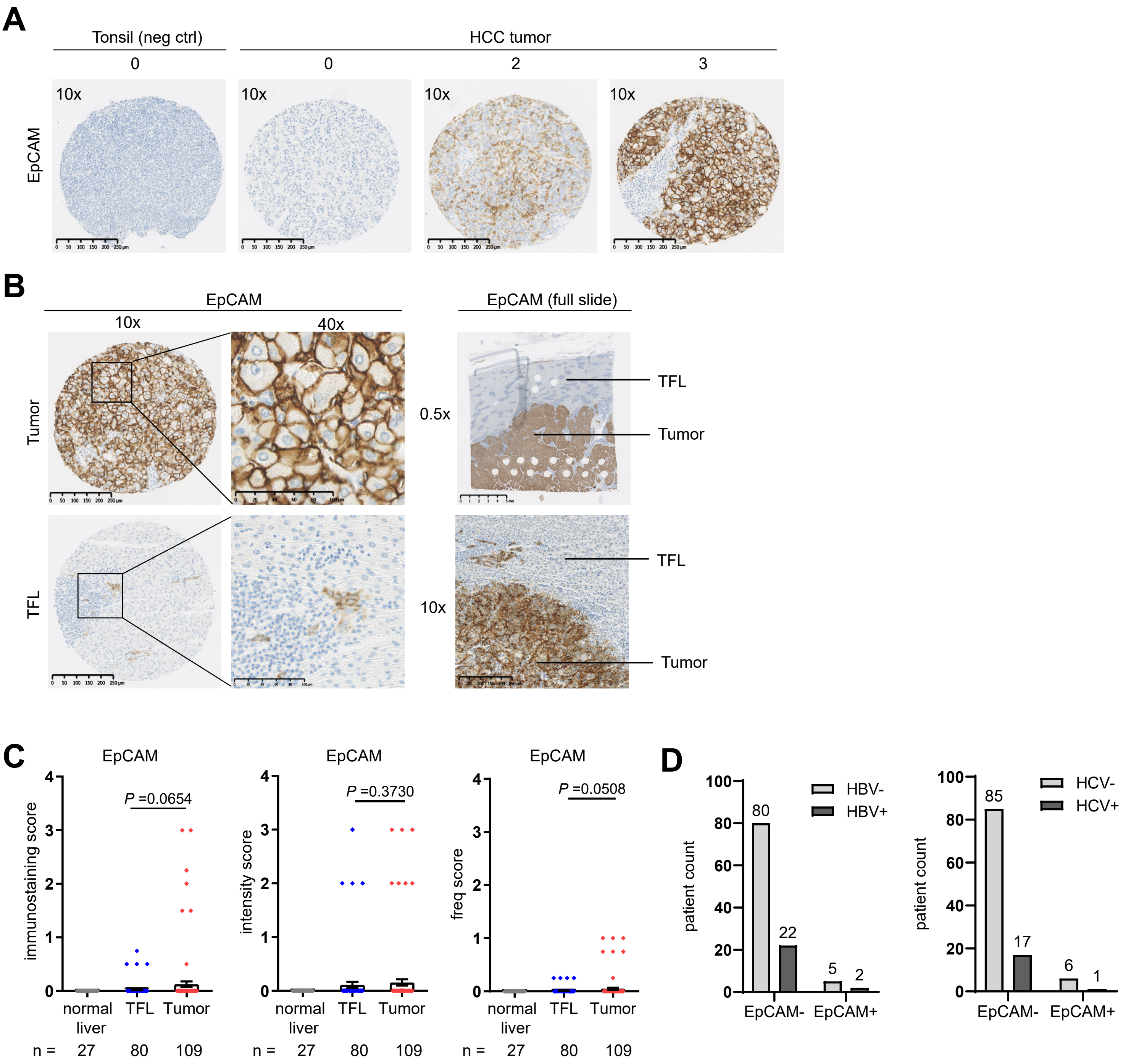

Fig. S1. EpCAM expression in HCC tumors

(A) Representative images of immunohistochemistry staining show EpCAM intensity scoring in tissue microarrays. Tonsil serves as negative control tissue. (B) Representative images of immunohistochemistry staining show EpCAM expression in HCC tumor and paired surrounding tumor-free liver (TFL) tissue. The immunostaining score for tumor is 3D and for TFL is 0. The full tissue section of this patient is also shown. Scale bars are presented in each image. (C) The immunostaining score, intensity score and frequency score of EpCAM in individual patients is presented (n=109). Significance was assessed by Wilcoxon matched-pairs signed rank test. *P*<0.05 is considered statistically significant. (D) The associations of EpCAM positivity of tumors with HBV/HCV infections are shown.

Supplementary Figure 2.

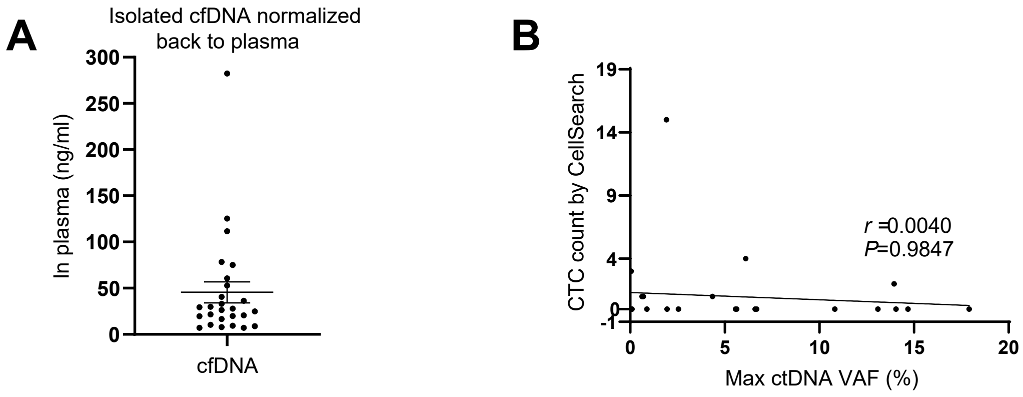

Fig. S2. CfDNA amount and the correlation between ctDNA VAF and CTC count

(A) The isolated cfDNA amount normalized to per ml plasma. (B) The correlation between CTC count and maximal ctDNA VAF.

Supplementary Table 1. An overview of ctDNA mutations and CTC count in advanced HCC patients

Patients	Total no. of mutations in cfDNA	Mutated Genes VAF (%)					Count
		TERT C228T	TP53	CTNNB1	PIK3CA	NRAS	EpCAM+ CTCs
1	1	17.92					0
2	2	6.61	3.61				0
3	1	6.11					4
4	1	5.57					0
5	2	5.39		5.63			0
6	4	4.35	0.29	1.73	0.35		1
7	2	3.95	10.82				0
8	3	3.87		14.68	13.99		0
9	2	3.67			13.09		0
10	1	2.55					0
11	1	1.95					0
12	1	1.92					15
13	2	1.50	14.05				0
14	1	0.88					0
15	1	0.69					1
16	2	0.63	0.28				1
17	2	0.35				6.68	0
18	1	0.09					0
19	2	0.09	13.95				2
20	1	0.05					3
21	0						0
22	0						0
23	0						0
24	0						0
25	0						0
26	0						0
	Total	20	6	3	3	1	7

Color scale: stronger color indicates higher frequency or number.

Supplementary Table 2. An overview of ctDNA mutation hotspots detected in HCC by NGS

Patient	Gene ID	Allele name	Mutation	VAF(%)	Chromosome location
2	TP53	p.R283P	C->G	3.61	Chr17_7577118
7	TP53	p.P278T	G->T	10.82	Chr17_7577118
13	TP53	p.R248W	G->A	14.05	Chr17_7577547
16	TP53	p.R249S	C->A	0.28	Chr17_7577547
19	TP53	p.R249S	C->A	13.95	Chr17_7577547
5	CTNNB1	p.T41A	A->G	5.63	Chr3_41266125
6	CTNNB1	p.T41A	A->G	1.73	Chr3_41266125
	PIK3CA	p.Q546R	A->G	0.35	SP_27.58329
	TP53	p.R282W	G->A	0.29	Chr17_7577118
8	CTNNB1	p.S45P	T->C	14.68	Chr3_41266125
	PIK3CA	p.Q546K	C->A	13.99	SP_27.58329
9	PIK3CA	p.E542K	G->A	13.09	SP_27.58329
17	NRAS	p.Q61K	G->T	6.68	SP_1.225761

Supplementary Table 3. Univariate and multivariate analysis of overall survival by Cox regression model

Clinicopathologic parameters	Univariate		Multivariate	
	HR (95% CI)	*P* value	HR (95%CI)	*P* value
ctDNA status: neg vs pos	5.382 (1.215-23.849)	0.027	3.996 (0.727-21.955)	0.111
CTC count: <2 vs ≥2	2.195 (0.679-7.093)	0.189		
Age, y: <60 vs ≥60	0.415 (0.156-1.102)	0.077		
Cirrhosis: yes vs no	0.499 (0.187-1.331)	0.165		
Tumor size:<10 vs ≥10	2.401 (0.685-8.416)	0.171		
Tumor number: 1 vs >1	0.578 (0.214-1.561)	0.280		
MVI: yes vs no	3.101 (1.111-8.654)	0.031	1.567 (0.489-5.023)	0.450
AFP: < 20 vs ≥20	1.586 (0.615-4.088)	0.340		

Supplementary Table 4. Comparison of genes in current study to Totoki's study[a]

gene	*Ge (n=26)*	*Totoki (n=452)*
TERT promoter	77%	55%
TP53	23%	31%
CTNNB1	12%	31%
PIK3CA	12%	1%
NRAS	4%	NA[b]
APC	0%	2%
BRAF	0%	NA
AKT1	0%	NA
EGFR	0%	NA
ERBB2	0%	NA
KRAS	0%	NA
GNAS	0%	NA
MAD4	0%	NA
MAP2K1	0%	NA
FBXW7	0%	<1%

a. Study represented here: Totoki et al. Nature Genetics 2014.
b. NA, not available. Because the frequency is not reported in the paper.

Supplementary Table 5: Torrent Variant Caller parameter settings

Parameter *	Value
snp_min_allele_freq	0.0005
snp_strand_bias	1
hotspot_min_coverage	3
sse_prob_threshold	1
try_few_restart_freq	1
hotspot_min_cov_each_strand	0
indel_min_var_coverage	2
hotspot_min_allele_freq	0.0005
report_ppa	0
mnp_min_variant_score	6
indel_func_size_offset	0
hotspot_strand_bias	1
filter_insertion_predictions	0.2
indel_min_variant_score	10
indel_min_coverage	3
heavy_tailed	3
snp_strand_bias_pval	0
outlier_probability	0.001
position_bias_ref_fraction	0.05
indel_strand_bias_pval	0
data_quality_stringency	20
snp_min_cov_each_strand	0
tag_sim_max_cov	10
indel_as_hpindel	0
hp_max_length	5
mnp_strand_bias	1
snp_min_coverage	3
use_fd_param	0
hotspot_min_var_coverage	2
mnp_strand_bias_pval	0
min_ratio_for_fd	0.1
hotspot_strand_bias_pval	0
hotspot_min_variant_score	3
max_flows_to_test	10
mnp_min_var_coverage	2
indel_strand_bias	1
position_bias	0.75
downsample_to_coverage	20000
filter_unusual_predictions	0.1
indel_min_allele_freq	0.0005
mnp_min_allele_freq	0.0005
mnp_min_coverage	3

mnp_min_cov_each_strand	0
fd_nonsnp_min_var_cov	1
tag_trim_method	sloppy-trim
prediction_precision	1
indel_min_cov_each_strand	0
filter_deletion_predictions	0.2
min_tag_fam_size	3
snp_min_variant_score	6
suppress_recalibration	0
position_bias_pval	0.05
use_position_bias	0
snp_min_var_coverage	2

* For detailed description of each parameter please read the Torrent Suite software user Guide 5.10:
https://assets.thermofisher.com/TFS-Assets/LSG/manuals/MAN0017598_TorrentSuiteSoftware_5_10_UG.pdf
